# Probiotics in CKD: waiting for the evidence

**DOI:** 10.1590/2175-8239-JBN-2023-E005en

**Published:** 2023-04-03

**Authors:** Letícia Fuganti Campos

**Affiliations:** 1 NUTROPAR, Curitiba, PR, Brazil.

The unquestionable role of the intestinal microbiota in health and the development of several diseases has resulted, in recent decades, in numerous publications on the subject. In parallel, interest in the use of probiotics to modulate the intestinal microbiota has intensified, resulting in an exponential increase in studies and publications that open frontiers for new treatments in several areas of medicine.

Evidence of a bidirectional relationship between dysbiosis and Chronic Kidney Disease (CKD) has also been reported. The intestinal microbiota interacts with kidney function through different complex mechanisms, including its influence on the immune system and on the formation of uremic toxins^1^. The so-called gut-brain-kidney axis links CKD to immune system dysregulation, metabolic disturbance, and sympathetic activation, and all these factors are linked to intestinal dysbiosis^2^. Crosstalk is achieved by the effects of dysbiotic metabolites on intestinal and kidney immunity, inflammation-induced intestinal barrier disruption, and the resulting influx of dysbiotic metabolites into the kidney through circulation^2^. Studies point out that intestinal modulation aimed at increasing short-chain fatty acid production and decreasing endotoxemia by lipopolysaccharides can delay kidney function deterioration, preventing or contributing to the treatment of CKD and its complications^1,3^.

In hemodialysis patients, results are divergent, and this therapy must be chosen with caution. The recent study published in BJN^4^ “Use of probiotics in patients with chronic kidney disease on hemodialysis: a randomized clinical trial” demonstrates the importance of better evaluating the effectiveness of probiotic therapy in patients on dialysis and sheds light on this knowledge.

We congratulate the authors for the excellent double-blind randomized clinical trial with 70 patients on hemodialysis, which showed that the use of probiotics (*Lactobacillus plantarum* A87, *Lactobacillus rhamnosus*, *Bifidobacterium bifidum* A218, and *Bifidobacterium longum* A101) for 3 months reduced the levels of syndecan-1 and glycemia, indicating possible improvements in metabolism and reduction in systemic inflammation. Syndecan-1 is one of the main components of the endothelial glycocalyx. Its expression in the immune system and its release in serum increases inflammatory stimuli, and this occur when there is damage to the endothelial glycocalyx, a condition present in CKD on hemodialytic therapy.

The finding of glycemic reduction was also extremely important, considering the close relationship between diabetes mellitus and CKD. Studies indicate that the use of probiotics improves glycemic control, especially when 3 or more strains of bacteria are associated^5^, as in the aforementioned study, which reinforces the potential of this treatment in this group of patients. Furthermore, studies show the importance of intestinal uremic toxins on inflammation as a significant cardiovascular risk factor, which is the main cause of death in dialysis patients. The study by Taki^6^ showed that the administration of *Bifidocteruim longum* reduced homocysteine levels in hemodialysis patients. However, the direct effect of probiotics in reducing cardiovascular mortality in this population has not yet been confirmed in randomized clinical trials^2^.

The risks of using probiotics in hemodialysis patients remain poorly explored. In the general population, probiotics could theoretically result in the following side effects: systemic infections, deleterious metabolic activities, excessive immune stimulation in susceptible individuals, and gene transfer. WHO/FAO recommend that new strains be evaluated for safety in human studies to assess side effects^7^. Thus, it is essential that the prescription of probiotics is always based on scientific evidence and individualized to the clinical condition of each patient.

Defining expected outcomes when using probiotics in patients on dialysis should also be an important factor to consider. The recently published study by Shamanadze et al showed that correction of intestinal flora with *L. acidophilu. B longum* and *S. Thermophilus* for a period of at least 12 weeks improved the quality of life of hemodialysis patients^8^. The choice of treatment based on scientific evidence must be aligned with the expected outcomes.

Finally, the definition of doses, the choice of strains, and the duration of use vary in the literature and remain uncertain, which makes it difficult to analyze the effectiveness of probiotics in patients on hemodialysis. Undoubtedly, the definition of these variables could contribute to the use of this tool in the challenging treatment of CKD. Furthermore, associating the use of probiotics with effective dietary interventions is essential, and future studies should consider this association^1^.
